# Bariatric surgery as a treatment of polycystic ovary syndrome: a systematic review and meta-analysis

**DOI:** 10.3389/fendo.2025.1682379

**Published:** 2026-01-05

**Authors:** Hyder Mirghani, Amani Shaman

**Affiliations:** 1 Internal Medicine Department, Faculty of Medicine, University of Tabuk, Tabuk, Saudi Arabia; 2 Department of Obstetrics and Gynecology Faculty of Medicine, University of Tabuk, Tabuk, Saudi Arabia

**Keywords:** bariatric surgery, ovarian hormones, menstruation, hypothalamic-pituitary, PCOS

## Abstract

**Background:**

Polycystic ovary syndrome (PCOS) is the most common endocrine disorder among females of reproductive age, and the majority are obese/overweight. PCOS management, including lifestyle and drugs, is limited by unsustainability and side effects. Bariatric surgery (BS) is promising in addressing hyperandrogenism and pregnancy outcomes. We aimed to assess the impact of bariatric surgery on PCOS components.

**Methods:**

We systematically searched PubMed/MEDLINE, Google Scholar, Cochrane Library, and Web of Science during July and August 2025, articles from inception up to August 2025 were included. The keywords used were BS, sleeve gastrectomy, gastric bypass, gastric banding, menstrual irregularities, free testosterone, total testosterone, hirsutism, SHBG, lutenizing hormone, antimullarian hormone (AMH), follicle-stimulating hormone, and pre-term deliveries. 648 articles were eligible, 35 full texts were reviewed, and 27 were included in the final meta-analysis.

**Results:**

Bariatric surgery reduced menstrual irregularities and hirsutism, with odds ratios of 27.68, 95% CI, 9.83-78.00, and 6.61, 95% CI, 0.97-47.07, respectively. In addition, total testosterone, free testosterone, AMH, and LH were reduced, SD, -19.95, 95% CI, -28.53–11.38, SD, 2.40, 95% CI, 1.30.53-3.51, SD, 1.66, 95% CI, 0.17-3.14, and SD, 2.21, 95% CI, 1.73-2.69 respectively, while SHBG were increased. No effects were observed regarding FSH, birth weight, gestational age, and pre-term delivery.

**Conclusion:**

BS reduced menstrual irregularities, hirsutism, total and free testosterone, AMH, and LH and increased SHBG. No significant differences were evident regarding other outcomes. Larger controlled trials investigating the long-term effects and the mechanism of action of BS on pregnancy outcomes, metabolic, and reproductive hormones are needed.

## Introduction

Polycystic ovary syndrome (PCOS) is the most common endocrine disorder in women of reproductive age, with a prevalence of 5% to 20% (1). The disease is multifactorial and extends throughout the female life from conception, and continues after the menopause (2). PCOS is diagnosed based on the presence of menstrual disturbances, hyperandrogenism, and polycystic ovaries. In addition, irregular menstrual cycles, oligo-anovulation, infertility, amenorrhea, and hirsutism are present (3).

There is continuous adaptation in the diagnostic criteria and interpretation of the pathophysiology of PCOS. However, there is a lack of uniform diagnostic criteria for the diagnosis and treatment (4). The available criteria include the National Institute of Health criteria, Rotterdam criteria, and AE-PCOS Criteria with four phenotypes described (5, 6), hyperandrogenism + ovulatory dysfunction + PCOM (phenotype A), hyperandrogenism + ovulatory dysfunction (phenotype B), hyperandrogenism + PCOM (phenotype C), and ovulatory dysfunction + PCOM (phenotype D). Importantly, other endocrine disorders with similar clinical manifestations, including thyroid disease, Cushing’s syndrome, and non-classical congenital adrenal hyperplasia, need to be ruled out (7, 8).

An efficient and timely diagnosis is mandatory for the implementation of treatment of PCOS and related comorbidities to improve the patient’s health and quality of life (6). The available management includes lifestyle (time-restricted feeding, high-intensity interval training, and ketogenic diet). Glucagon-like peptide-1 receptor agonists (GLP-1RAs) alone or with metformin are effective in metabolic and reproductive outcomes. Other therapies include statins, vitamin D, spironolactone, clomiphene citrate, cyproterone acetate, and oral contraceptive pills. The above medications address specific symptoms/clinical pictures. However, they are associated with many unwanted effects, including weight gain, gastrointestinal side effects, hepatotoxicity, and mood swings (9–11). Because of that, a treatment that can address most of the PCOS with acceptable side effects is highly needed.

Bariatric surgeries are shown to reduce weight, improve fertility, hirsutism, and metabolic comorbidities in women with PCOS. More effective (12, 13). Bariatric surgery was shown to be more effective compared to metformin alone in obese women with PCOS, and women with PCOS and infertility should consider bariatric surgery for better pregnancy rates and menstrual irregularity (14). Meta-analyses investigating the effects of bariatric surgery on PCOS clinical and hormonal factors are scarce. Yue et al. (15) investigated the effects of bariatric surgery on menstrual irregularities, testosterone, hirsutism, and body mass index, and showed the positive impact of bariatric surgery. Tian showed a reduction of total testosterone, lutenizing hormone, and glycemic parameters with increasing estrogen. However, follicle-stimulating hormone (FSH) and LH/FSH were not affected (16), and Chen et al. (17) observed a reduction in body mass index, testosterone, ovarian volume, and menstrual irregularities. The above meta-analyses were limited by the small number of included studies, the high heterogeneity, and did not cover all the components of PCOS. Therefore, an update is justifiable. Because of that, we conducted this meta-analysis in which we aimed to assess the effects of bariatric surgery on menstrual irregularities, free and total testosterone, hirsutism, AMH, sex, SHBG, LH, FSH, and pre-trem deliveries in women with PCOS.

## Subjects and methods

This meta-analysis was conducted in July and August 2025 with strict adherence to the PRISMA Guidelines.

### Inclusion criteria

Clinical trials, retrospective, prospective, and case-control studies were included; the studies should measure the effect of different bariatric surgeries on PCOS components.

### Exclusion criteria

Commentaries, opinions, letters to the Editor, case reports, and study protocols were excluded.

### Population

All women with polycystic ovaries in the reproductive age who undergo bariatric surgery.

### Intervention

All types of bariatric surgery, including sleeve gastrectomy, gastric bypass, and banding.

### Outcome measures

The outcome measures were the effect of bariatric surgery on menstrual irregularities, free and total testosterone, hirsutism, SHBG, LH, HSH, and pre-trem deliveries in women with PCOS.

### Literature search

We systematically searched PubMed/MEDLINE, Google Scholar, Cochrane Library, and Web of Science for relevant articles that assessed the effects of bariatric surgery on the different components of PCOS and pre-term delivery. The literature search was conducted during July and August 2025, and articles from inception up to August 2025 were included The keywords used were bariatric surgery, sleeve gastrectomy, gastric bypass, gastric banding, menstrual irregularities, free testosterone, total testosterone, hirsutism, antimullerian hormone, sex hormone binding globulin, lutenizing hormone, follicle-stimulating hormone, and pre-term deliveries. Six hundred and forty-eight articles were eligible, and 547 remained after duplication removal. Of them, 35 full texts were reviewed, and twenty-seven full texts were included in the final meta-analysis.

### Data extraction

**Figure 1:**
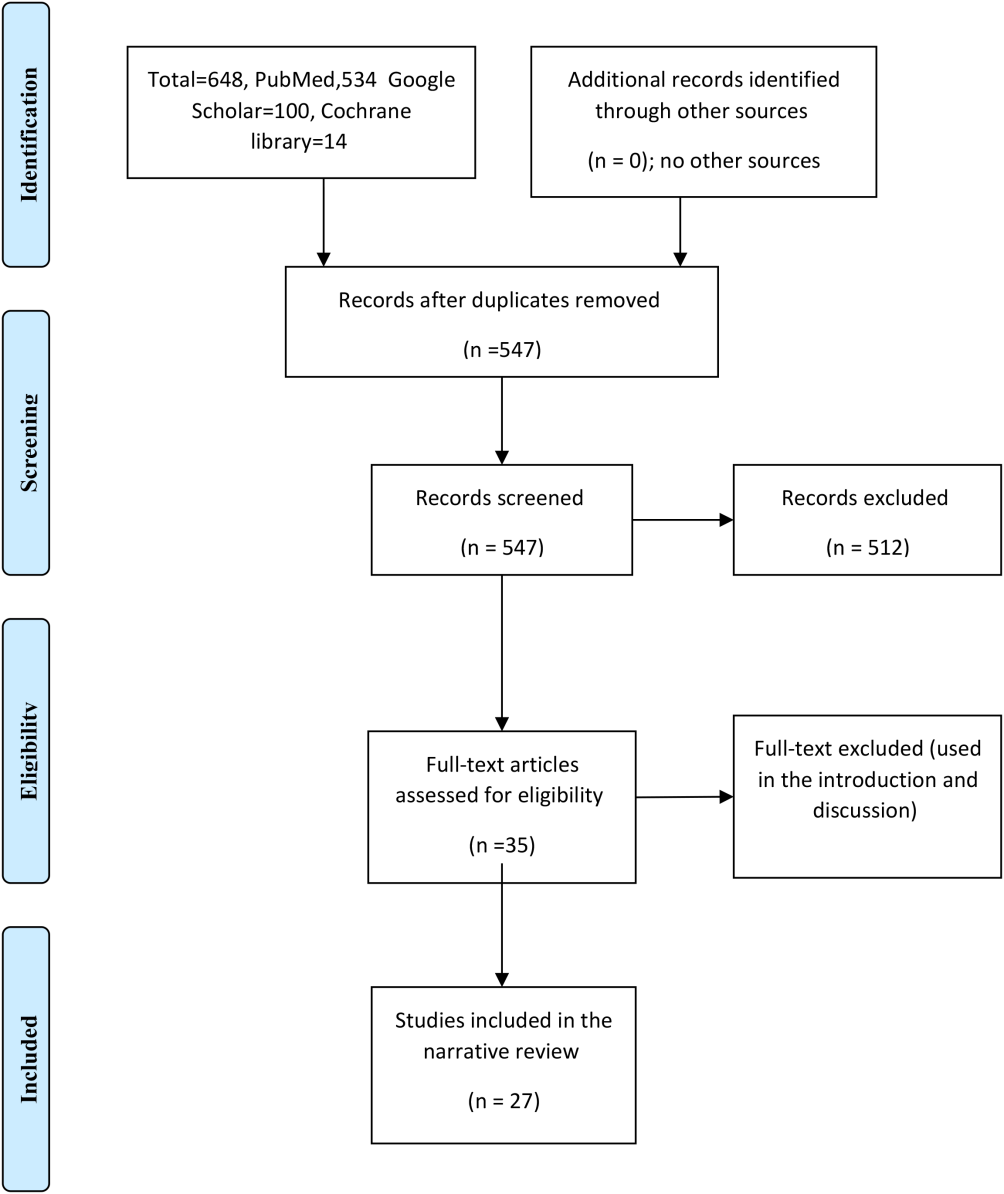
Studies investigating the effects of bariatric surgery in women with polycystic ovary syndrome (The PRISMA Chart).

The author’s name, publication year, country, study type, study duration, number of participants, body mass index, and type of bariatric surgery were entered in an Excel sheet. In addition, we recorded free testosterone, total testosterone, hirsutism, AMH, SHBG, LH, FSH, and pre-trem deliveries before and after surgery. **Figure 1**, **Tables 1**–**3**.

**Table 1:** Basic characteristics of the included studies.

Author	Study type	Country	Age	BMI/pre/post-surgery	Study duration/months	Number of patients	Preterm delivery pre-/post-surgery	Bariatric surgery
Alamdari et al., 2024 (19)	Prospective	Iran	31.69 ± 9.54	44.28 ± 3.03 vs. 29.37 ± 2.4	12	50	Not assessed	LSG
Alhumaidan et al., 2024 (20)	Cross-sectional	Saudi Arabia	18-50	–	–	516	Not assessed	LSG in the majority
Bhandari et al., 2022 (21)	Prospective	India	31.03 ± 0.23	43.6 ± 7.2	6	1013	Not assessed	LSG
Cai et al., 2023 (22)	Retrospective	China	28.9 ± 1.63	38.65 ± 1.65 Vs. 28.64 ± 2.1	38	88	Not assessed	LSG
Casals et al., 2021 (23)	Retrospective	Spain	33.19 ± 4.91	45.59 ± 4.97 Vs. 27.73 ± 4.34	10.1 ± 1.5	43	Not assessed	LSG, LRYGB
Christ et al., 2018 (24)	Retrospective	USA	36.1 ± 2.3	44.2 ± 2.1 Vs. 35.4 ± 1.5	22.8 ± 3.6	44	Not assessed	Not specified
Eid et al., 2014 (25)	Retrospective	USA	36.3 ± 8.4	44.8 ± 5.9 Vs. 29.2 ± 5.9	12	14	Not assessed	RGBY
Escobar-Morreale et al., 2005 (26)	Prospective	Spain	29.8 ± 5.3	50.7 ± 7.1 Vs. 41 ± 9	12	17	Not assessed	BPD, RYGB
Gamal et al., 2012 (27)	Retrospective	USA	32 ± 5.8	52.8 ± 9.08	46.7	20	Not assessed	RGBY
Lacey et al., 2023 (28)	Prospective	UK	Not mentioned	Not mentioned	12	77	Not assessed	–
Li et al., 2025 (29)	Prospective	China	29 ± 5.65	40.6 ± 5.88 Vs. 25.73 ± 2.44	12	192	Not assessed	Not mentioned
Singh et al., 2020 (30)	Prospective	India	29.7 ± 5.9	44.9 ± 7.5	12	18	Not assessed	LSG, OAGB, gastric bypass
Tammo et al., 2025 (31)	Retrospective	Turkey	29.4 ± 5.21	42.9 ± 3.47 vs. 31.8 ± 2.52	34	72	3/17 versus 1/27	LSG, Banding
Turkmen et al., 2016 (32)	Prospective	Sweden	29.92 ± 7.12	47.15 ± 7.57 Vs. 35.46 ± 7.04	6	13	Not assessed	RYGB
Wang et al., 2015 (33)	Prospective	China	25 ± 9.19	35.2 ± 11.8 Vs. 31.7 ± 2.8	6	24	Not assessed	LSG
Wang et al., 2023 (34)	Prospective	China	33.78 ± 7.31	41.22 ± 6.35 Vs. 28.98 ± 5.52	12	24	Not assessed	LSG
Zhao et al., 2024 (35)	Retrospective	China	28.68 ± 0.35	40.31 ± 6.14	12	229	Not assessed	LSG
Christinajoice et al., 2020 (36)	Retrospective	India	24.7 ± 10.2	41.5 ± 6.8	36	29	Not assessed	LSG, RYGB, LAGB
Benito et al., 2020 (37)	Retrospective	Spain	32.1 ± 5.3	44.6 ± 4.7 Vs. 28.5 ± 4.1a	19	49	Not assessed	LSG, RGBY, Banding
Ezzat et al., 2021 (38)	Prospective	Egypt	27.2 ± 4.21	43.6 ± 1.76 Vs. 29.1 ± 1.17	12	36	Not assessed	LSG, gastric bypass
Chofalo et a. 2017 (39)	Retrospective	Italy	30 ± 6	44 ± 7	12	29	Not assessed	SG, RYGB
Hu et al., 2022 (40)	Prospective	China	28.7 ± 0.7	35.6 ± 4.24 Vs. 23.7 ± 2.82	12	41	Not assessed	LSG
Samarasinghe et al., 2024 (41)	Trial	UK	30 ± 6	45 ± 6.64	12	40	Not assessed	LSG
Nayak et al., 2023 (42)	Retrospective	India	28.88 ± 4.0	40.83 ± 3.65 vs 29.33 ± 4.12	12	17	Not assessed	Not specified.
Anbara et al., 2023 (43)	Prospective	Iran	31.6 ± 6.8	39.6 ± 7.8 Vs.	6	23	Not assessed	LSG
Hochberg et al., 2024 (44)	Retrospective	Canada	25-35	35-40	24	141	19/141 versus 1798/14741	RYGB
Huke et al., 2024 (45)	Retrospective	Norway	23.2 ± 5.1	46.1 ± 6.2 vs. 37.7 ± 2.3	36	35	0/13versus 2/35	RYGB

**Table 2:** Menstrual irregularities, hirsutism, testosterone levels, sex hormone binding globulins, antimullerian hormone, follicle-stimulating and luteinizing hormones before and after bariatric surgery.

Author	Abnormal menstruation/post-surgery	Hirsutism	Total testesteron/per-post Surgery/ng/dL	Free testesterone/per-post Surgery/ng/dL	Sex hormone binding globulin/pre-post Surgery/nmol/L	AMH/per-post Surgery/ng/ml	FSH/per-post Surgery/mlU/ml	LH/per-post Surgery/mlU/ml
Alamdari et al., 2024 (19)	50/50 versus 17/50	Not assessed	Not assessed	Not assessed	Not assessed	Not assessed	Not assessed	Not assessed
Alhumaidan et al., 2024 (20)	216/516 versus 187/516	Not assessed	Not assessed	Not assessed	Not assessed	Not assessed	Not assessed	Not assessed
Bhandari et al., 2022 (21)	28/43 versus 12/43	993/1013 versus 262/1013	Not assessed	1.6 ± 1.4 versus 0.5 ± 0.2	Not assessed	4.68 ± 1.85 versus 3.38 ± 1.21	Not assessed	Not assessed
Cai et al., 2023 (22)	83/88 versus 78/83	Not assessed	47.01 ± 8.1 versus 36.63 ± 9.23	11.75 ± 7.15 Versus 2.24 ± 1.56	18.98 ± 5.25 versus 61.8 ± 26.98	Not assessed	Not assessed	Not assessed
Casals et al., 2021 (23)	28/43 versus 7/43	Not assessed	Not assessed	Not assessed	Not assessed	Not assessed	Not assessed	Not assessed
Christ et al., 2018 (24)	37/44 versus 7/44	Not assessed	45.9 ± 6.9 Versus 19.7 ± 5.9	0.9 ± 0.9 versus 0.4 ± 0.7	Not assessed	Not assessed	Not assessed	Not assessed
Eid et al., 2014 (25)	4/14 versus 0/14	11/14 versus 7/14	59 ± 8.2 versus 33.7 ± 4.4	5.9 ± 0.2 versus 2.2 ± 0.26	Not assessed	Not assessed	Not assessed	Not assessed
Escobar-Morreale et al., 2005 (26)	12/16 versus 0/12	Not assessed	73 ± 33 versus 42 ± 19	1.6 ± 0.7 versus 0.6 ± 0.3	Not assessed	Not assessed	Not assessed	Not assessed
Gamal et al., 2012 (27)	17/20 versus 3/17	14/20 versus 10/20	Not assessed	Not assessed	Not assessed	Not assessed	Not assessed	Not assessed
Lacey et al., 2023 (28)	52/77 versus 27/77	49/77 versus 15/77	Not assessed	Not assessed	Not assessed	Not assessed	Not assessed	Not assessed
Li et al., 2025 (29)	171/187 versus 16/84	Not assessed	49.61 ± 20.48 versus 37.49 ± 10.38	Not assessed	Not assessed	3.7 ± 2.02 Versus 3.83 ± 2.31	5.54 ± 1.26 Versus 5.43 ± 1.47	8.13 ± 2.91 Versus 5.95 ± 3.2
Singh et al., 2020 (30)	18/18 versus 0/18	16/18 versus 6/11	83 ± 38 versus 42.1 ± 25	Not assessed	Not assessed	Not assessed	Not assessed	Not assessed
Tammo et al., 2025 (31)	46/72 versus 29/72	Not assessed	34.1 ± 13.4 versus 25.1 ± 7.15	Not assessed	61.2 ± 55.8 Versus 123.7 ± 37.9	4.52 ± 1.43 Versus 4.16 ± 1.26	Not assessed	Not assessed
Turkmen et al., 2016 (32)	13/13 versus 6/13	Not assessed	56.4 ± 17.3 versus 31.2 ± 13.8	Not assessed	25.88 ± 14.77 versus 48.43 ± 23.99	Not assessed	Not assessed	Not assessed
Wang et al., 2015 (33)	24/24 versus 3/24	Not assessed	56.2 ± 7 versus 31 ± 10	Not assessed	Not assessed	Not assessed	Not assessed	Not assessed
Wang et al., 2023 (34)	27/32 versus 0/32	Not assessed	Not assessed	Not assessed	Not assessed	Not assessed	4.8 ± 1.6 Versus 5.8 ± 2.5	6.1 ± 2.0 Versus 4.4 ± 1.9
Zhao et al., 2024 (35)	229/229 versus 48/229	Not assessed	Not assessed	Not assessed	Not assessed	Not assessed	Not assessed	Not assessed
Christinajoice et al., 2020 (36)	Not assessed	11/29 versus 10/29	Not assessed	Not assessed	Not assessed	Not assessed	Not assessed	Not assessed
Hu et al., 2022 (40)	Not assessed	Not assessed	57.68 ± 26.53versus 37.49 ± 20.48	Not assessed	15.1 ± 0.56 Versus 62.3 ± 1.61	9.3 ± 1.15 Versus 6 ± 0.71	Not assessed	Not assessed
Samarasinghe et al., 2024 (41)	Not assessed	Not assessed	51.91 ± 10.1 Versus 34.61 ± 12.11	Not assessed	30 ± 6.9 versus 51.8 ± 7.42	31.2 ± 11.17 Versus 23.7 ± 11.73	5.3 ± 1.7 Versus 5.3 ± 9.2	9.2 ± 2.2 Versus 6.4 ± 2.7
Benito et al., 2020 (37)	Not assessed	Not assessed	69.16 ± 28.82 Versus 43.23 ± 17.29	1.38 ± 0.75 versus 0.6 ± 0.35	49 ± 63 Versus 89 ± 84	Not assessed	Not assessed	Not assessed
Ezzat et al.2021 (38)	Not assessed	Not assessed	36.7 ± 8.5 Versus 20.4 ± 4.7	5.15 ± 1.65Versus 3.43 ± 1.11	16.3 ± 2.31versus 29.3 ± 4.21	Not assessed	Not assessed	Not assessed
Cai et al., 2023 (22)	Not assessed	Not assessed	47.01 ± 8.1 versus 36.63 ± 9.23	11.75 ± 7.15 Versus 2.24 ± 1.56	18.98 ± 5.25versus 61.8 ± 26.98	Not assessed	Not assessed	Not assessed
Nayak et al., 2023 (42)	Not assessed	Not assessed	Not assessed	Not assessed	Not assessed	4.77 ± 4.71 Versus 3.12 ± 0.64	Not assessed	Not assessed
Chofalo et al., 2017 (39)	Not assessed	Not assessed	Not assessed	Not assessed	Not assessed	5.44 ± 3.74 versus 4.25 ± 3.65	Not assessed	Not assessed
Anbara et al., 2023 (43)	Not assessed	Not assessed	Not assessed	Not assessed	Not assessed		7.8 ± 2.5 Versus 11.6 ± 3.5	5.2 ± 1.8 Versus 6.8 ± 2.3

### Quality assessment of the included studies

The quality of the included studies was assessed using the Methodological Index for non-randomized studies (minors) (18). The index has 13 components each with a score of 2 and a total maximum score of 26. The scores of the included studies ranged from 10-26. **Table 4**.

**Table 3:** The quality of the included studies according to the Methodological Index for non-randomized studies (minors).

Study	A clearly stated aim	Inclusion of consecutive patients	Prospective collection of data	Endpoint appropriate to the aim of the study	Unbiased assessment of the study endpoint	Follow-up period appropriate to the aim	Loss to follow-up < 5%	Prospective calculation of the study size	An adequate control group	Contemporary groups	Baseline equivalence of groups	Adequate statistical analyses	Total score
Alamdari et al., 2024 (19)	2	2	2	2	2	1	2	0	0	0	1	2	16
Alhumaidan et al., 2024 (20)	2	2	2	2	1	2	0	0	N. C	N. C.	N. C	N. C	11
Bhandari et al., 2022 (21)	2	2	2	2	2	0	1	0	2	2	1	2	18
Cai et al., 2023 (22)	2	2	0	2	2	2	2	0	2	2	2	2	20
Casals et al., 2021 (23)	2	2	2	2	2	2	0	0	2	1	1	2	18
Christ et al., 2018 (24)	2	2	2	1	1	2	1	0	N. C	N. C.	N. C	N. C	11
Eid et al., 2014 (25)	2	2	1	1	2	2	0	0	N. C	N. C.	N. C	N. C	10
Escobar-Morreale et al., 2005 (26)	2	2	2	2	2	2	0	0	1	2	1	2	18
Gamal et al., 2012 (27)	2	2	1	1	2	2	0	0	N. C	N. C.	N. C	N. C	10
Lacey et al., 2023 (28)	2	2	1	1	2	2	0	0	N. C	N. C.	N. C	N. C	10
Li et al., 2025 (29)	2	2	2	2	2	2	0	2	N. C	N. C.	N. C	N. C	14
Singh et al., 2020 (30)	2	2	1	1	2	2	0	0	N. C	N. C.	N. C	N. C	10
Tammo et al., 2025 (31)	2	2	0	2	2	2	2	0	2	2	1	2	19
Turkmen et al., 2016 (32)	2	2	1	1	1	1	2	0	N. C	N. C.	N. C	N. C	10
Wang et al., 2015 (33)	2	2	2	2	2	1	0	0	2	2	1	2	18
Wang et al., 2023 (34)	2	2	1	2	2	2	2	2	N. C	N. C.	N. C	N. C	15
Zhao et al., 2024 (35)	2	2	2	2	2	0	1	0	2	2	1	2	18
Christinajoice et al., 2020 (36)	2	2	2	2	1	2	0	0	N. C	N. C.	N. C	N. C	11
Benito et al., 2020 (37)	2	2	2	2	2	2	0	0	2	2	2	2	20
Ezzat et al., 2021 (38)	2	2	0	2	2	2	2	0	0	2	0	2	16
Chofalo et a. 2017 (39)	2	2	1	2	2	2	2	0	2	2	1	2	20
Hu et al., 2022 (40)	2	2	2	2	2	2	2	2	2	2	2	2	26
Samarasinghe et al., 2024 (41)	2	2	2	2	2	2	0	2	2	2	1	2	23
Nayak et al., 2023 (42)	2	2	0	2	2	2	2	0	2	2	2	2	20
Anbara et al., 2023 (43)	2	2	0	2	2	2	2	0	2	2	2	2	20
Hochberg et al., 2024 (44)	2	2	0	2	2	2	2	0	2	0	0	2	16
Huke et al., 2024 (45)	2	2	2	2	2	1	0	0	2	2	1	2	18

### Statistical analysis

We used the Cochrane tool for meta-analysis (RevMan, version 5.4, Oxford, United Kingdom) for data analysis, the dichotomas and continuous data were manually entered and the data were analyzed, odds ratio, and standard differences were estimated at 95% confidence interval, the heterogeneity was assessed and considered significant when *I*^2^ was ≥ 50% and the random effect was used, the fixed effect was used for *I*^2^ < 25%. Forest plots and funnel plots were generated, the Chi-Square test was assessed with mean differences, and Z Scores. A subgroup analysis to remove studies with significant heterogeneity. A p-value< 0.05 was considered significant.

## Results

### Characteristics of the included studies

We included 27 studies (19–45) (13 prospective, 12 retrospective, one trial, and one cross-sectional), 14 studies were published in Asia, 8 in Europe, one in Canada, one in Africa, and 3 in the USA.

In the present meta-analysis, we included 17 studies (19–35) and 1796 patients With 1466 events and found that menstrual irregulatary reduced significantly following different bariatric surgery procedures, odds ratio, 27.68, 95% CI, 9.83-78.00, a significant heterogeneity was found, I^2^ = 93%, Chi-Square=231.00, P-value for heterogeneity <0.001, Z score=6.28, standard difference=16, and P-value for overall effect < 0.001.

**Figure 2:**
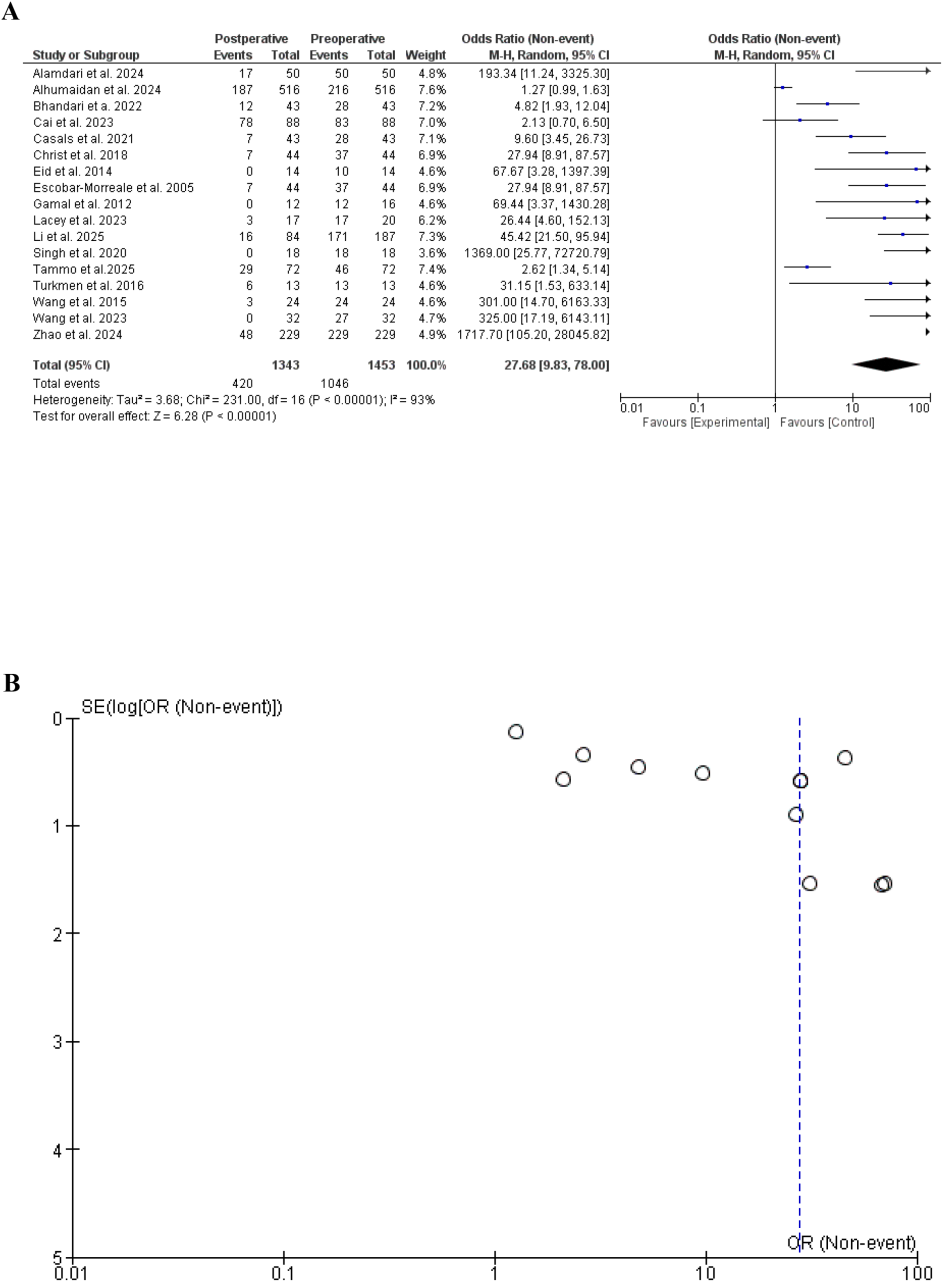
**(A)** Menstrual irregularities before and after bariatric surgery in women with polycystic ovary syndrome. (Forest plot). **(B)** Menstrual irregularities before and after bariatric surgery (Funnel plot). **(A)** Menstrual irregularities before and after bariatric surgery in women with polycystic ovary syndrome. (Forest plot after removing studies with high heterogeneity).

The results were not changed after the removal of the studies with significant contribution to heterogeneity, odds ratio, 22.21, 95% CI, 12.71-38.81, no significant heterogeneity was found, I^2^ = 0%, Chi-Square=4.04, P-value for heterogeneity, 0.67, Z score=10.89, standard difference=6, and P-value for overall effect < 0.001. **Figures 2A, B**.

Bariatric surgery was shown to reduce hisrsutism in women with PCOS (21, 25, 27, 28, 30. 36), odds ratio, 6.61, 95% CI, 0.97-47.07, a significant heterogeneity was found, I^2^ = 96%, Chi-Square=119.19, P-value for heterogeneity <0.001, Z score=1.93, standard difference=5, and P-value for overall effect, 0.05.

**Figure 3:**
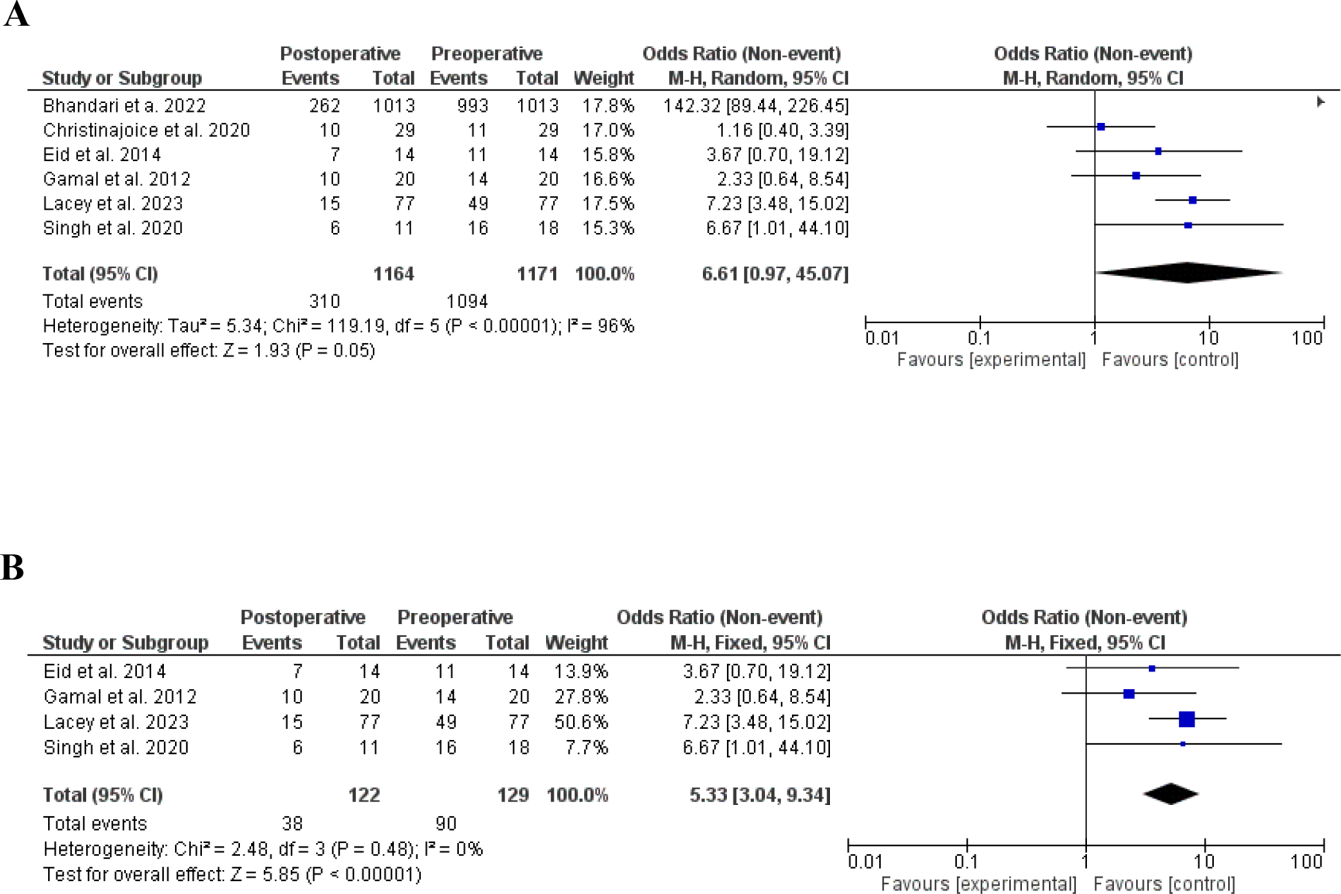
**(A)** Hirsutism before and after bariatric surgery in women with polycystic ovary syndrome. **(B)** Hirsutism before and after bariatric surgery in women with polycystic ovary syndrome, no heterogeneity.

The results were not changed after removing studies with significant contribution to heterogeneity, odds ratio, 5.33, 95% CI, 3.04-9.34, no significant heterogeneity was found, I^2^ = 0%, Chi-Square=2.48, P-value for heterogeneity, 0.48, Z score=5.85, standard difference=3, and P-value for overall effect <0.001. **Figures 3A, B**.

**Figure 4:**
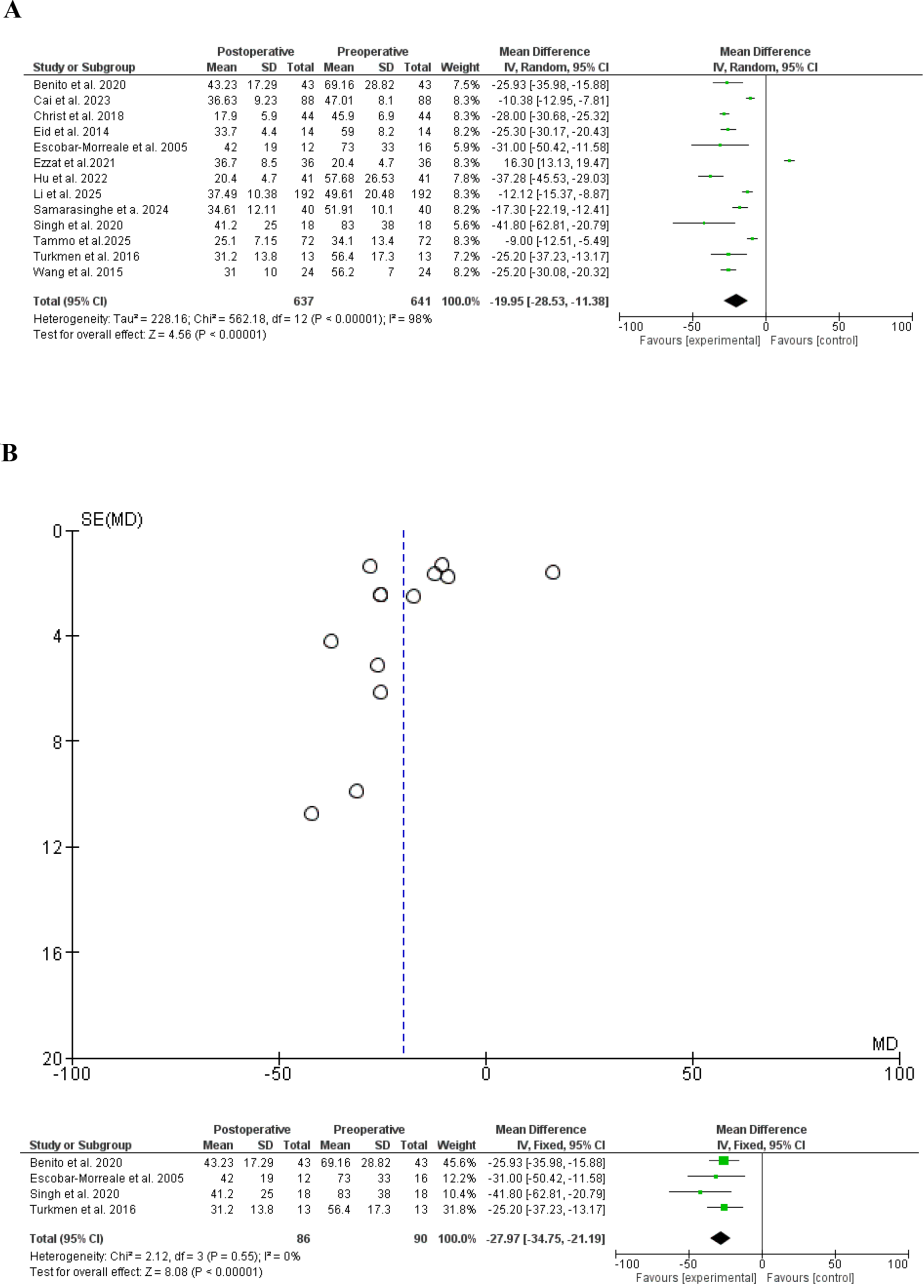
**(A)** Total testosterone before and after bariatric surgery in women with polycystic ovary syndrome. **(B)** Total testosterone before and after bariatric surgery in women with polycystic ovary syndrome (Funnel plot). **(B)** Total testosterone before and after bariatric surgery in women with polycystic ovary syndrome, no heterogeneity.

The effects of bariatric surgery on total testesterone was assessed in 13 studies (22, 24–26, 29–33, 37, 38, 40, 41) in which bariatric surgery significantly reduced the total testesterone, standard difference (SD), -19.95, 95% CI, -28.53–11.38, a significant heterogeneity was found, I^2^ = 98%, Chi-Square=562.18, P-value for heterogeneity <0.001, Z score=4.56, standard difference=12, and P-value for overall effect < 0.001. The results remained robust after eliminating heterogeneity, SD, -27.97, 95% CI, -34.75–21.19, no significant heterogeneity was found, I^2^ = 0%, Chi-Square=562.18, P-value for heterogeneity, 0.55, Z score=8.08, standard difference=3, and P-value for overall effect < 0.001. **Figures 4A, B**.

**Table 4:** Birth weight, gestational age, and pre-term birth in women with PCOS before and following bariatric surgery.

Author	Study type	Birth weight/PCOS	Birth weight/control	Gestational age		Preterm/low birth weight	Preterm /low birth weight
Benito et al., 2020 (37)	Retrospective	2763 ± 618/17	3155 ± 586/27	38.1 ± 3.2	39.2 ± 2.7	3	1
Huke et al., 2024 (45)	Retrospective	3396±526	3490±538	39.1±1.7	39.4±1.8	2	7
Hochberg et al., 2024 (44)	Retrospective	Not assessed	Not assessed	Not assessed	Not assessed	19/141	1798/ 14741

**Figure 5:**
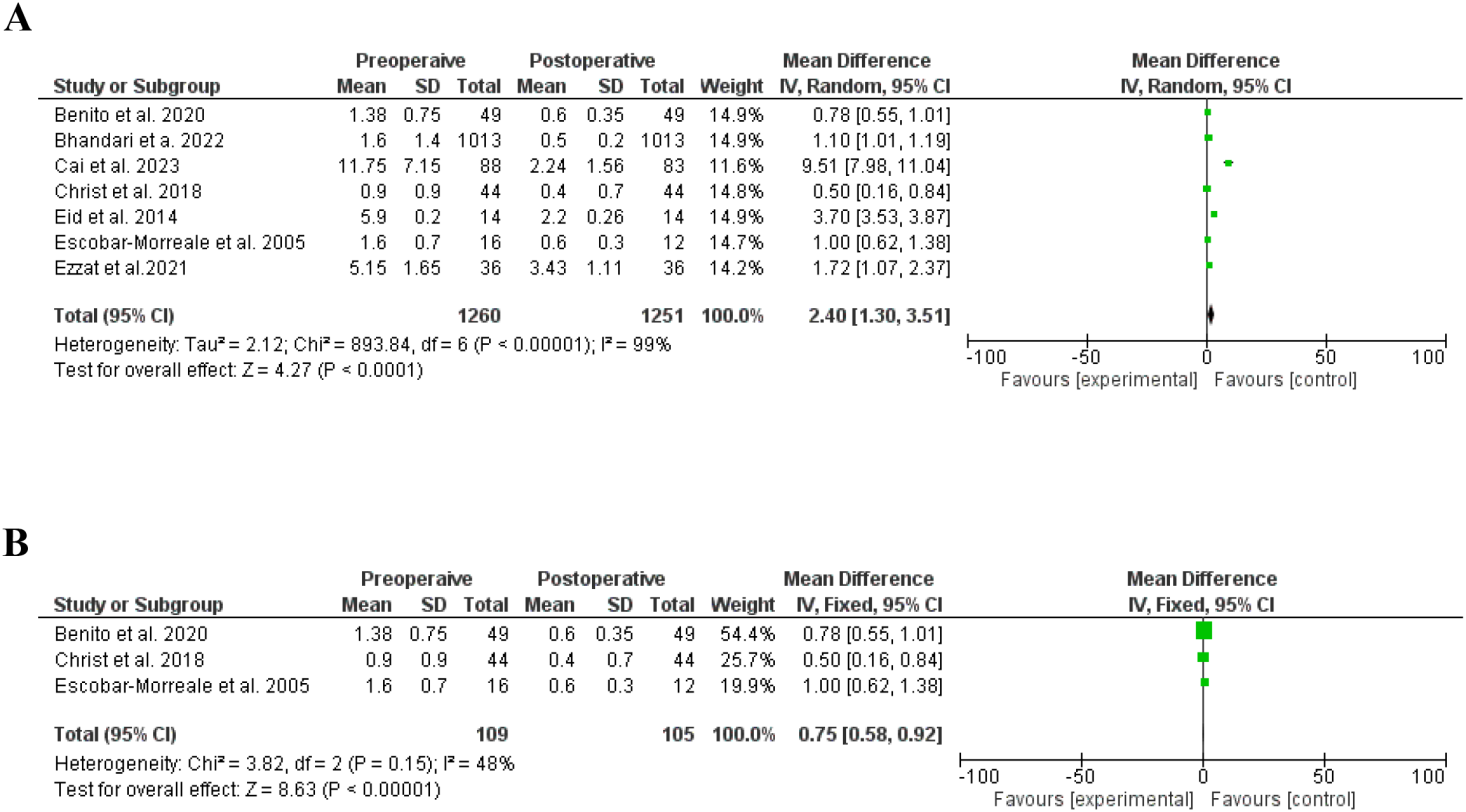
**(A)** Free testosterone before and after bariatric surgery in women with polycystic ovary syndrome. **(B)** Free testosterone before and after bariatric surgery in women with polycystic ovary syndrome, no significant heterogeneity.

Similarly, bariatric surgery significantly reduced the free testesterone (21, 22, 24–26, 37, 38, 41, 42), SD, 2.40, 95% CI, 1.30.53-3.51, a significant heterogeneity was found, I^2^ = 99%, Chi-Square=893.84, P-value for heterogeneity <0.001, Z score=4.27, standard difference=6, and P-value for overall effect < 0.001. The results were not different without significant heterogeneity, SD, 0.75, 95% CI, 0.58-0.92, no significant heterogeneity was found, I^2^ = 48%, Chi-Square=3.82, P-value for heterogeneity, 0.15, Z score=8.63, standard difference=2, and P-value for overall effect < 0.001. **Figures 5A, B**.

**Figure 6:**
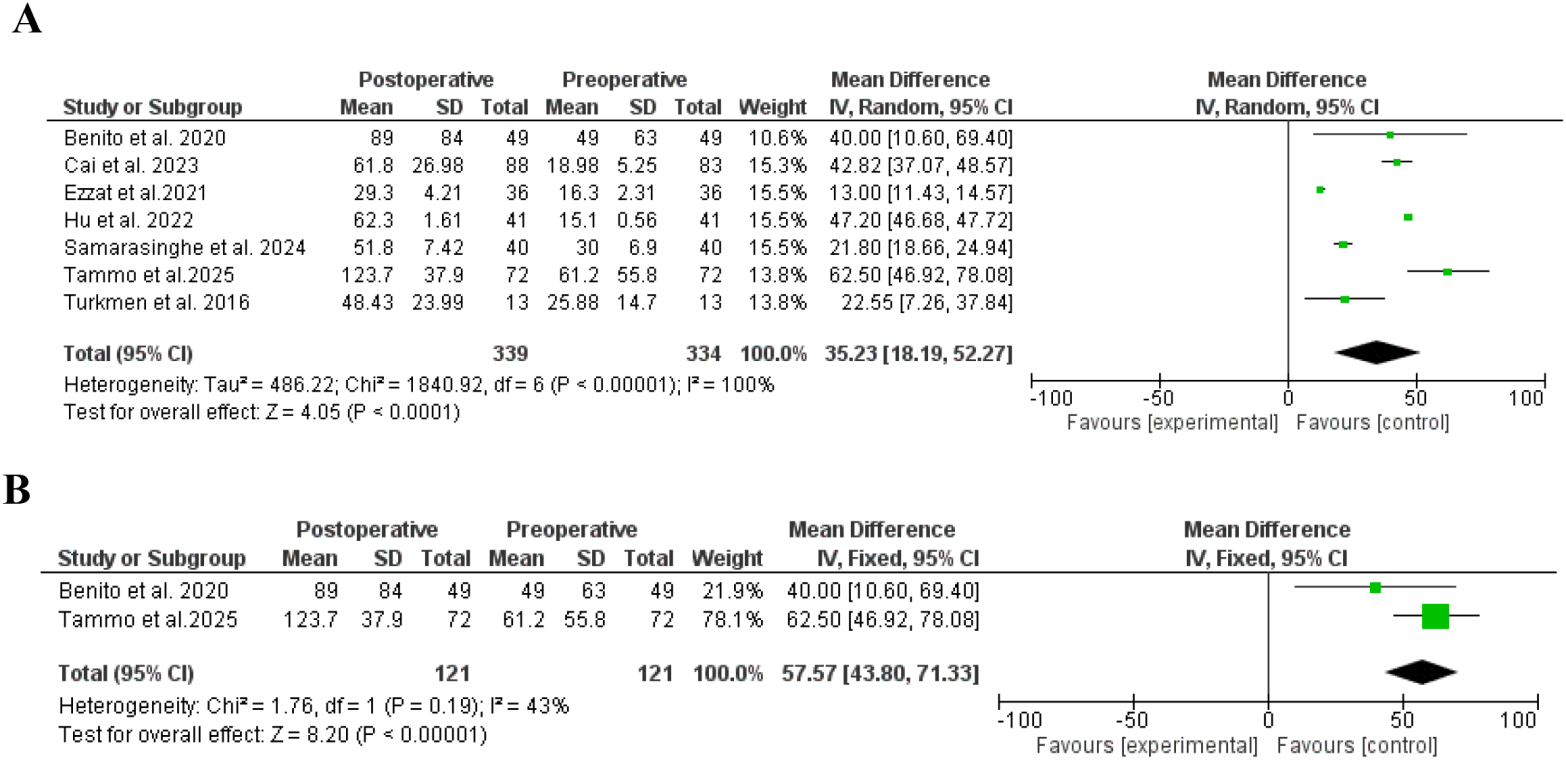
**(A)** Sex hormone binding globulin before and after bariatric surgery in women with polycystic ovary syndrome. **(B)** Sex hormone binding globulin before and after bariatric surgery in women with polycystic ovary syndrome, no significant heterogeneity.

Sex hormone binding globulin increased significantly following bariatric surgery (21, 22, 31, 32, 37, 38, 40, 41), SD, 35.23, 95% CI, 18.19-52.27, a significant heterogeneity was found, I^2^ = 100%, Chi-Square=1840.92, P-value for heterogeneity <0.001, Z score=4.05, standard difference=6, and P-value for overall effect < 0.001. The results were the same after including studies without heterogeneity, SD, 57.57, 95% CI, 43.80-71.33, no significant heterogeneity was found, I^2^ = 43%, Chi-Square=1.76, P-value for heterogeneity, 0.19, Z score=8.20, standard difference=1, and P-value for overall effect < 0.001. **Figures 6A, B**.

Regarding the effects of bariatric surgery on ovarian hormones, AMH reduced significantly following bariatric surgery (21, 29, 31, 39–42), SD, 1.66, 95% CI, 0.17-3.14, a significant heterogeneity was found, I^2^ = 97%, Chi-Square=154.91, P-value for heterogeneity <0.001, Z score=2.18, standard difference=5, and P-value for overall effect < 0.001.

**Figure 7:**
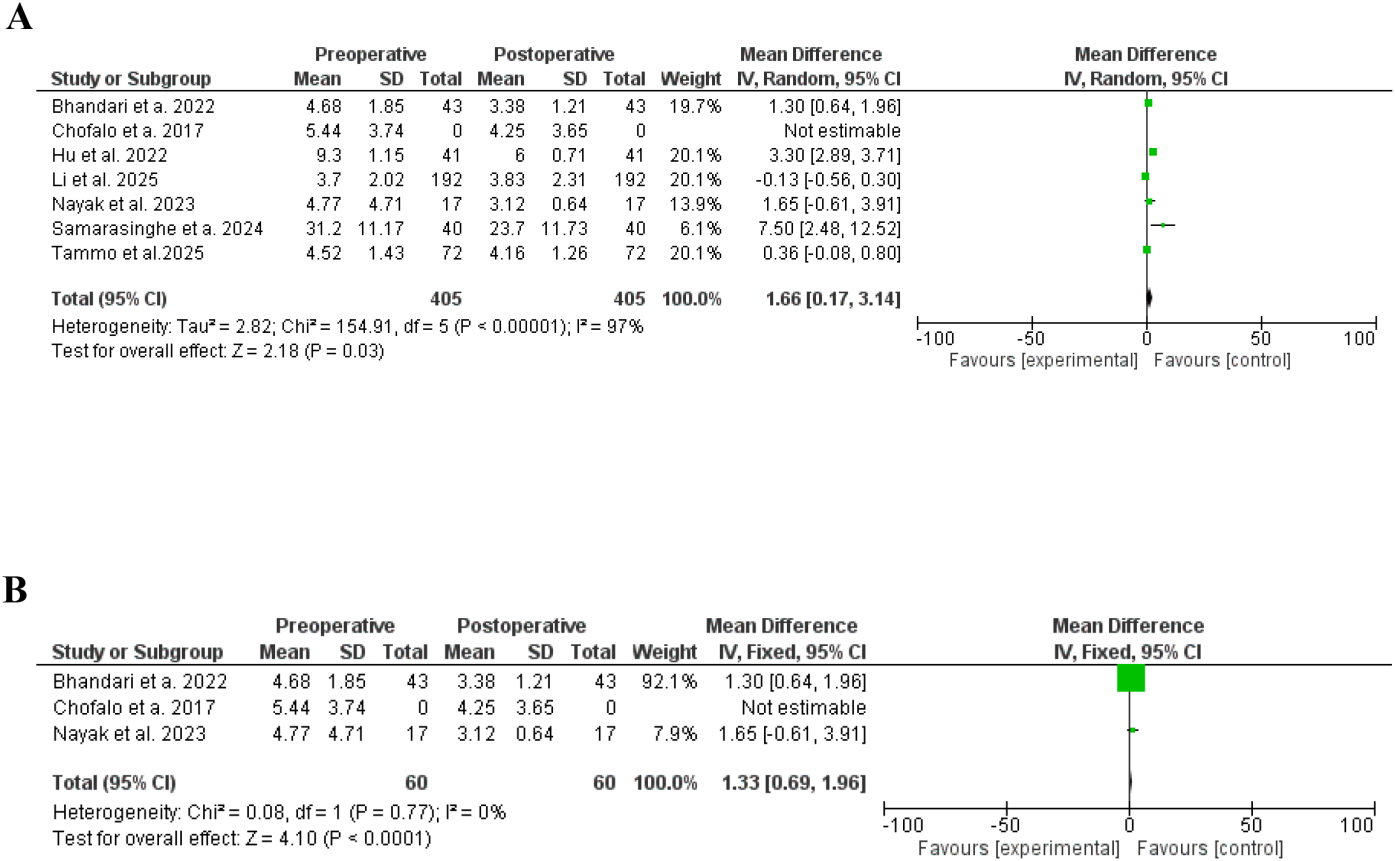
**(A)** Antimullerian hormone before and after bariatric surgery in women with polycystic ovary syndrome. **(B)** Antimullerian hormone before and after bariatric surgery in women with polycystic ovary syndrome, no significant heterogeneity.

The results remained significant after removing studies with high heterogeneity, SD, 1.33, 95% CI, 0.69-1.96, no significant heterogeneity was found, I^2^ = 0%, Chi-Square=0.08, P-value for heterogeneity, 0.77, Z score=4.10, standard difference=1, and P-value for overall effect < 0.001. **Figures 7A, B**.

In this meta-analysis, LH, and FSH were not affected by bariatri surgery (29, 34, 41, 43), SD, 1.30, 95% CI, 0.36-2.97, a significant heterogeneity was found, I^2^ = 92%, Chi-Square=35.71, P-value for heterogeneity <0.001, Z score=1.54, standard difference=3, and P-value for overall effect, 0.12, and SD, -1.15, 95% CI, -2.80-0.51, a significant heterogeneity was found, I^2^ = 88%, Chi-Square=21.24, P-value for heterogeneity <0.001, Z score=1.36, standard difference=3, and P-value for overall effect, 0.17 respectively.

**Figure 8:**
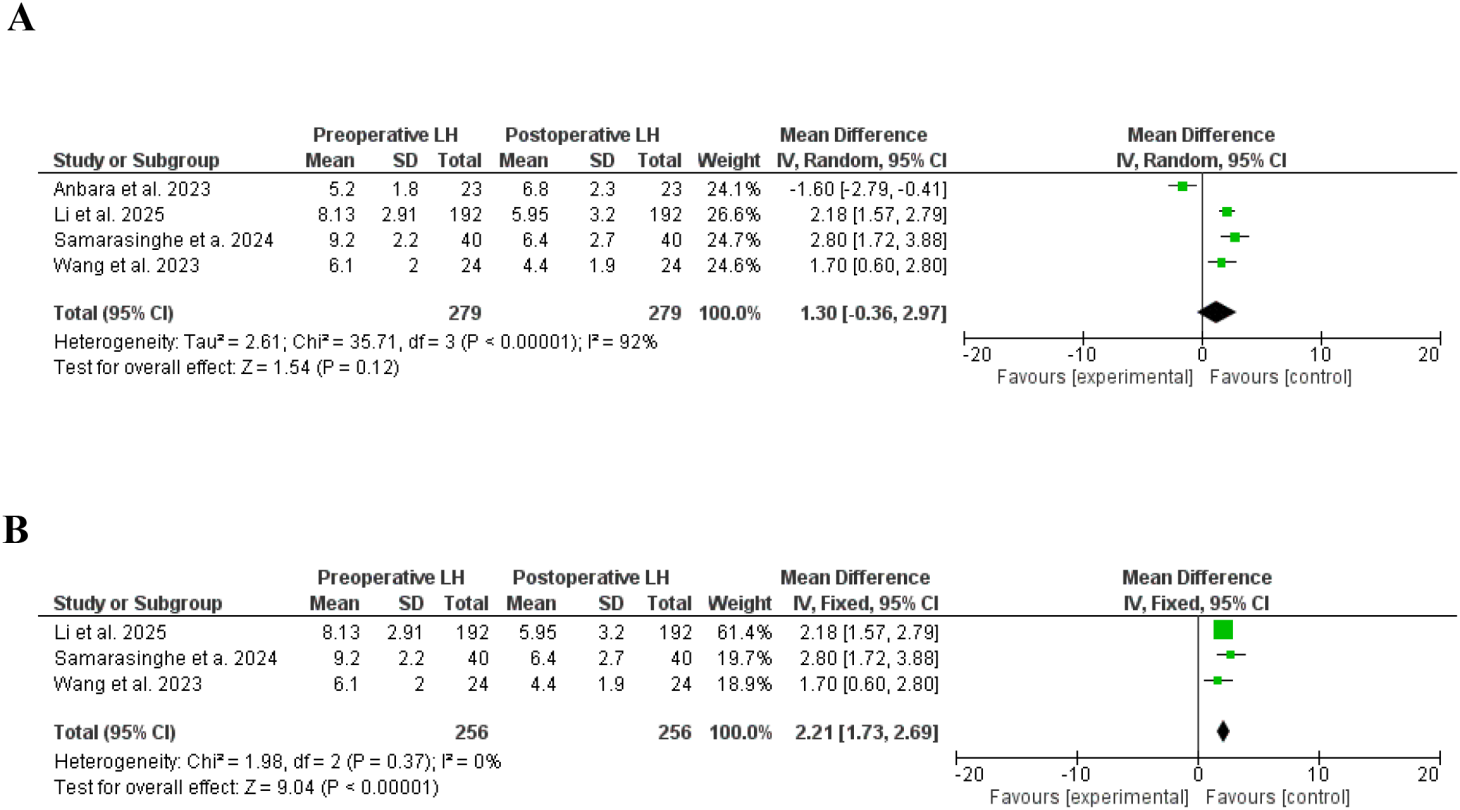
**(A)**. Lutenizing hormone before and after bariatric surgery in women with polycystic ovary syndrome. **(B)** Lutenizing hormone before and after bariatric surgery in women with polycystic ovary syndrome, no significant heterogeneity.

**Figure 9:**
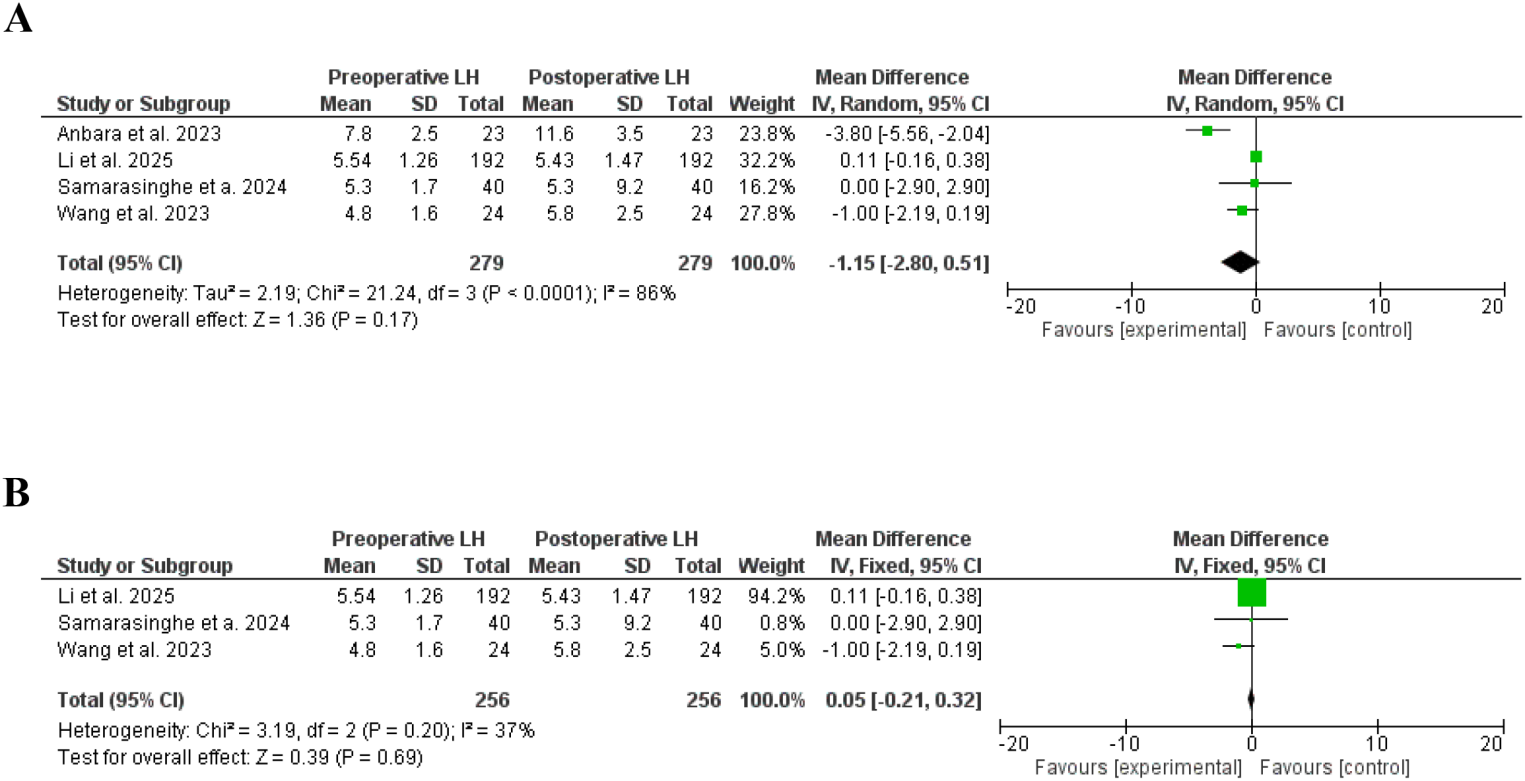
**(A)** Follicular-stimulating hormone before and after bariatric surgery in women with polycystic ovary syndrome. **(B)** Follicular-stimulating hormone before and after bariatric surgery in women with polycystic ovary syndrome, no significant heterogeneity.

Importantly, LH was significantly lower after removing studies with significant heterogeneity, SD, 2.21, 95% CI, 1.73-2.69, no significant heterogeneity was found, I^2^ = 0%, Chi-Square=1.98, P-value for heterogeneity, 0.37, Z score=9.04, standard difference=2, and P-value for overall effect < 0.001. However, the FSH levels were not changed after removing studies with high heterogeneity, SD, -0.05, 95% CI, -0.21-0.32, no significant heterogeneity was found, I^2^ = 37%, Chi-Square=3.19, P-value for heterogeneity, 0.20, Z score=0.39, standard difference=2, and P-value for overall effect, 0.67. **Figures 8A, B**, **9A, B**.

**Figure 10:**
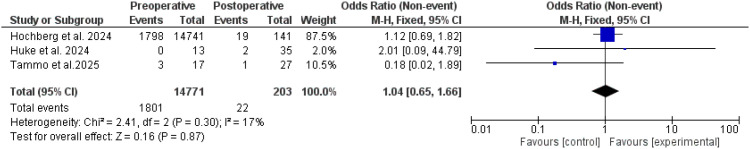
Pre-term delivery before and after bariatric surgery in women with polycystic ovary syndrome.

In this meta-analysis, we assessed the pre-term delivery before and after bariatric surgery and found no differences, odds ratio, 1.04, 95% CI, 0.65-1.66, no significant heterogeneity was found, I^2^ for heterogeneity=17%, Chi-Square=2.41, and P-value for heterogeneity, 0.30, Z score=0.16, standard difference=2, and P-value for overall effect, 0.87. **Figure 10**.

## Discussion

PCOS is common (19.9%), with hyperandrogenism and polycystic ovaries being the commonest phenotype. Importantly, obesity is prevalent in patients with PCOS and ranges from 50% to 80%. Obesity in PCOS is mediated by hypothalamo-pituitary imbalance and leads to polycystic ovaries through various mechanisms. In addition, obesity-mediated inflammation and oxidative stress negatively impact reproductive function in women with PCOS (46, 47).

In the present study, all the patients were obese/overweight. Obesity leads to insulin resistance, hyperinsulinism, lipogenesis, and decreases lipolysis, sensitizes ovarian follicles to huetinizing hormone effects, and upregulates androgen production by the ovaries (48). The reproductive, endocrine, and metabolic disorders are usually triggered by obesity in patients with a susceptible genetic background (49). Therefore, obesity management is vital to restore fertility and address the metabolic and endocrine function in women with PCOS.

Lifestyle modifications and drug therapy for PCOS are limited by the transient efficacy, while bariatric surgery is the most promising intervention (12). Chen et al. (17) conducted a meta-analysis and included nine studies; they found a reduction in menstrual irregularity, hypertrichosis, and free testosterone levels in line with our results. However, the authors could not assess the SHBG, AMH, and pregnancy and fertility outcomes. We assessed the effect of bariatric surgery on SHBG and observed an increasing levels (SD, 35.23, 95% CI, 18.19-52.27) with a reduction in AMH (SD, 1.66, 95% CI, 0.17-3.14), AMH is a predictor of ovarian reserve and its increasing level as following bariatric surgery highlighted the importance of bariatric surgery in improving the chance of pregnancy and life birth (50). Our findings were similar to Yue et al. (16), who found a reduction in abnormal menstruation, hirsutism, total and free testosterone, AMH, and increasing SHBG. However, their findings were limited by the small number of included studies. In the current study, we included 27 high-quality studies to give a broader insight into the effects of bariatric surgery on the PCOS components.

The pathogenesis of anovulation and infertility is mediated by insulin resistance and obesity. Obese women develop hyperinsulinemia, hyperandrogenism, and hypothalamic-pituitary-ovarian axis dysfunction. In addition, high androgens impair follicular growth and maturation, leading to sparse ovulation, abnormal menstruation, and hirsutism (51, 52). Another important finding in this study is the increasing levels of SHBG following bariatric surgery. Women with PCOS had low levels of SHBG, which binds testosterone and reduces its levels, ameliorating its unwanted effects, including metabolic syndrome, type 2 diabetes, and cardiovascular disease (53, 54). AMH is an indicator of ovarian reserve, and low levels are predictors of early pregnancy loss and could be a biomarker of oocyte competence (55); therefore, the current findings of AMH reduction following bariatric surgery could improve fertility and decrease pregnancy loss in women with PCOS.

In this meta-analysis, we found no differences between pre-term delivery before and after bariatric surgery (odds ratio, 1.04, 95% CI, 0.65-1.66), similarly, the available studies found no significant differences between women with PCOS and their counterparts without the syndrome regarding birth weight and gestational age respectively. Arbis et al. (56) conducted a meta-analysis and showed no significant impact of bariatric surgery on gestational age or birth weight in line with the current findings. However, Akhter and colleagues (57) found higher rates of preterm birth, small-for-gestational age, mortality, and congenital anomalies in women following bariatric surgeries; the reasons behind the complications were nutritional deficiencies essential for fetal development following bariatric surgery (58). The contradicting results could be explained by the differences in bariatric surgeries (restrictive, malabsorptive, and combined) and the differences in the basic characteristics of the included patients.

To the best of our knowledge, this is the first meta-analysis to assess birth weight, gestational age, and pre-term delivery in women with PCOS following bariatric surgery. The literature on this important topic is scarce, and the current recommendations are the use of long-acting reversible contraception before bariatric surgery and continued for 12 months (59). The mechanisms through which bariatric surgery improves PCOS are undetermined: weight loss and restoring insulin sensitivity could explain the neuroregulation of the hypothalamic-pituitary axis and hyperandrogenism (60, 61). However, the improvement in menstrual irregularities observed shortly after bariatric surgery cannot be explained by weight loss alone (25); gut microbiota disruption, bile acids, and other gut hormone disturbances following bariatric surgery could explain the findings (62). In this meta-analysis, LH, and FSH were not affected by bariatri surgery, SD, 1.30, 95% CI, 0.36-2.97, and SD, -1.15, 95% CI, -2.80-0.51, our finding were similar to Tian et al. (16) regarding FSH, however, they found a reduction in LH, the contradiction could be explained by the differences in the included studies. We found a reduction in LH levels after removing studies with high heterogeneity in similarity to Tan et al. (findings).

Shorter gestations, an increased risk of small-for-gestational-age following bariatric surgery were reported by Johansson et al. (63), and a meta-analysis that included 13 studies (64). However, the studies were not conducted in women with PCOS. In the present meta-analysis, no significant differences were evident regarding birth weight and gestational age in women with PCOS before and after bariatric surgery (37, 45). This result imply that bariatric surgery might not negatively impact fetal outcomes. Further studies are needed to solve the issue.

The strength of this study is that we included recently published 13 studies (19, 20, 22, 28, 29, 31, 34, 35, 40, 41, 43–45), and assessed pre-term delivery, gestational age, and birth weight, which were not studied by the previous meta-analysis.

### The study limitations

This meta-analysis was limited by the observational studies included and the high heterogeneity observed.

## Conclusion

Bariatric surgery reduced menstrual irregularities, hirsutism, total and free testosterone, and antimullerian hormone, and increased SHBG. No significant differences were evident regarding FSH, LH, birth weight, gestational age, and pre-term delivery. Larger controlled trials investigating the long-term effects of bariatric surgery on pregnancy outcomes and the mechanism through which bariatric surgery acts in women with PCOS are needed.

## Data Availability

The original contributions presented in the study are included in the article/supplementary material. Further inquiries can be directed to the corresponding author.
